# Migrant men and HIV care engagement in Johannesburg, South Africa

**DOI:** 10.1186/s12889-024-17833-2

**Published:** 2024-02-12

**Authors:** Maria Francesca Nardell, Caroline Govathson, Sithabile Mngadi-Ncube, Nkosinathi Ngcobo, Daniel Letswalo, Mark Lurie, Jacqui Miot, Lawrence Long, Ingrid Theresa Katz, Sophie Pascoe

**Affiliations:** 1 Division of Global Health Equity, Brigham and Women’s Hospital, 75 Francis Street, Boston, MA 02115 USA; 2 Harvard Medical School, Boston, MA USA; 3 Health Economics and Epidemiology Research Office (HE2RO), Johannesburg, South Africa; 4 Faculty of Health Sciences, University of the Witwatersrand, Johannesburg, South Africa; 5 Brown University School of Public Health, Brown University, Providence, Rhode Island, USA; 6 Department of Global Health, Boston University School of Public Health, Boston, MA USA; 7 Division of Women’s Health, Brigham and Women’s Hospital, Boston, MA USA

**Keywords:** Migration, Mobility, Men, HIV Care, HIV Testing, HIV Prevention

## Abstract

**Background:**

South Africa (SA) has one of the highest rates of migration on the continent, largely comprised of men seeking labor opportunities in urban centers. Migrant men are at risk for challenges engaging in HIV care. However, rates of HIV and patterns of healthcare engagement among migrant men in urban Johannesburg are poorly understood.

**Methods:**

We analyzed data from 150 adult men (≥ 18 years) recruited in 10/2020–11/2020 at one of five sites in Johannesburg, Gauteng Province, SA where migrants typically gather for work, shelter, transit, or leisure: a factory, building materials store, homeless shelter, taxi rank, and public park. Participants were surveyed to assess migration factors (e.g., birth location, residency status), self-reported HIV status, and use and knowledge of HIV and general health services. Proportions were calculated with descriptive statistics. Associations between migration factors and health outcomes were examined with Fisher exact tests and logistic regression models. Internal migrants, who travel within the country, were defined as South African men born outside Gauteng Province. International migrants were defined as men born outside SA.

**Results:**

Two fifths (60/150, 40%) of participants were internal migrants and one fifth (33/150, 22%) were international migrants. More internal migrants reported living with HIV than non-migrants (20% vs 6%, *p* = 0.042), though in a multi-variate analysis controlling for age, being an internal migrant was not a significant predictor of self-reported HIV positive status. Over 90% all participants had undergone an HIV test in their lifetime. Less than 20% of all participants had heard of pre-exposure prophylaxis (PrEP), with only 12% international migrants having familiarity with PrEP. Over twice as many individuals without permanent residency or citizenship reported “never visiting a health facility,” as compared to citizens/permanent residents (28.6% vs. 10.6%, *p* = 0.073).

**Conclusions:**

Our study revealed a high proportion of migrants within our community-based sample of men and demonstrated a need for HIV and other healthcare services that effectively reach migrants in Johannesburg. Future research is warranted to further disaggregate this heterogenous population by different dimensions of mobility and to understand how to design HIV programs in ways that will address migrants’ challenges.

**Supplementary Information:**

The online version contains supplementary material available at 10.1186/s12889-024-17833-2.

## Introduction

South Africa has one of the highest rates of migration on the continent [1]. In urban Gauteng Province alone, an estimated 29% of the population is “internal” migrants who have changed residences within the country and 6% is international migrants who have crossed country borders [2]. Migration and mobility, both within and into South Africa, have been key drivers of the country’s HIV epidemic [3, 4]. While there are growing numbers of migrant women [5], the majority of migrants are men seeking work in urban centers [6, 7]. These men are often driven by a lack of employment opportunities [8] and shifts in climate affecting agricultural practices in rural areas [9–11], and they often retain strong ties to their homes of origin [8]. Migrant men in South Africa have borne twice the burden of HIV compared to non-migrant men with an HIV prevalence of ~ 25.9% [12]. Migrant men have been shown to be less likely to engage in HIV services, including HIV testing [13–16], pre-exposure prophylaxis (PrEP) [17, 18], and antiretroviral therapy (ART) [19, 20].

The associations between migration and HIV acquisition, care engagement, and HIV-related outcomes are complex and incompletely understood [5, 21]. Some research has shown that people already living with HIV are more likely to migrate [22, 23]. Other evidence shows that some migrants may be healthier when they leave their homes of origin, often called the “healthy migrant” hypothesis [24]. Migrants often have worse mental and physical health outcomes in their new destinations [21, 25]. Individuals who travel more frequently away from home have been shown to be at higher risk for acquiring HIV than those who travel less [26]. In addition, migration and mobility pose significant challenges to health systems which assume a stable catchment area. South Africa’s decentralized health system is ill-suited for facilitating people changing clinics because it is difficult to easily transfer patient data across facilities [27]. Migrants also face other intersecting psychosocial and structural challenges which affect HIV and healthcare engagement, such as the disruption of relocation [22], lifestyle changes [5], unstable or lack of employment [21], isolation and difficult living conditions [25], lack of residency or citizenship documentation [28], and fear and language issues [29]. While some migrants nonetheless seek care in South Africa’s public healthcare system [30], research suggests that international migrants may also access healthcare at sites that are community-based or religiously-affiliated, especially where there are no fees or requirements for identity documentation or residence status [31]. For male migrants, challenges in seeking and accessing care are compounded by internal and social constructs of masculinity, which impede care engagement in healthcare settings felt to be oriented towards women’s needs [32].

The heterogenous nature of population mobility and varying definitions of migration further complicate efforts to understand migration and healthcare engagement, especially across different settings and populations [33, 34]. For example, some studies focus on the temporal nature of mobility (e.g., seasonal migration [35]), while others focus on specific social reasons for mobility (e.g., market traders [36] or miners [37]), or spatial aspects of mobility, such as how distance traveled affects HIV acquisition risk [38, 39]. In addition, there are challenges accessing migrant men in research. For example, large trials to improve HIV testing uptake among men in South Africa have found substantial barriers to reaching men in communities with high mobility patterns [40]. A demographic surveillance study from rural South Africa showed that 69% of the adult population cohort migrated out of the study area at least once during a 13 year follow-up period [22]. Most of these men seek employment opportunities in cities in Gauteng Province, where Johannesburg is located, and yet research describing migrants within these urban destination sites remains limited and often comes from ethnographic rather than HIV studies [41, 42].

Movement into and within South Africa, particularly from rural to urban centers, continues to rise along with the country’s rapid socio-economic growth [22] and accelerating urbanization throughout the region [43]. There is a growing recognition of the need to make HIV care more accessible to migrants in order to achieve UNAIDS 95–95-95 goals [32]. Yet despite migrants’ vulnerability to HIV acquisition [39, 44] and susceptibility to poor health engagement [20, 45, 46], there are few recent studies that seek to describe urban South African migrants, document their rates of HIV, or understand their health seeking behaviors [47, 48]. In this study, we sought to understand these gaps by characterizing migrant men within a broader sample of men recruited at community venues in Johannesburg, Gauteng Province, for a study to understand men’s patterns of care engagement. Gauteng Province has the largest concentration of people living with HIV in South Africa, including an average HIV prevalence of 14.7% for men [49]. In this study, we aimed to analyze participants’ burden of HIV, rates of HIV testing, awareness and knowledge of PrEP, and overall healthcare utilization. We hypothesized that both internal and international migrants, compared to non-migrants, would be at a greater risk for acquiring HIV, and be less likely to engage in treatment and prevention services. Lastly, we hypothesized that migrants would be less likely than non-migrants to have ever visited a health facility, and that migrants without citizenship or permanent residency would be less likely to have ever visited a health facility, as compared to citizens or permanent residents.

## Methods

This cross-sectional, migration-specific study was embedded within our team’s broader “Men’s Choice” study, which included a prospective cohort of 150 adult men recruited at community-based, non-healthcare sites in Johannesburg in October and November 2020. The goal of the “Men’s Choice” study was to understand men’s preferences for accessing HIV and other healthcare services in Johannesburg through a discrete choice experiment (DCE) survey [50]. The results of the DCE have been presented elsewhere [50, 51]. For this study, we administered a survey that preceded the DCE survey and included questions on men’s migration patterns, HIV care utilization, and socio-demographic measures.

### Participants, study setting, and procedures

Five Johannesburg recruitment sites in the Hillbrow, Midrand, Woodmead and Roodeport areas were chosen following a period of observation and discussion with community stakeholders. These sites (factory, building materials store, taxi rank, homeless shelter, and public park) were selected to represent a range of locations where men go for work, transit, shelter, and leisure. We chose to focus on sites outside of healthcare facilities in order to reach men who may not seek care at traditional healthcare settings. Recruitment occurred during the Covid-19 pandemic-related restrictions. Therefore, we used a convenience sampling method in which team members recruited available and interested participants by directly distributing study flyers at some sites (taxi rank, public park) or by asking leadership at some sites (homeless shelter, factory site, building materials store) to distribute flyers on the team’s behalf. These flyers gave information about the study and invited men to contact the study team if they were interested in participating. Those men who made contact were then given further information and taken through the consent process over the phone. The team checked telephone numbers, names, and identifiers to ensure that the same participant did not enroll twice. The study was conducted in English, isiZulu, Setswana, isiXhosa, and Sesotho. Several of the first survey interviews were completed over the phone, but as restrictions lifted in later 2020, survey interviews were then completed face-to-face. After completing the survey, participants were reimbursed for their time with a ZAR150 (~ USD10) electronic shopping voucher, sent directly to the participant’s cell phone number.

### Eligibility

We included men who were 18 years or older, willing to participate in a 2-h survey (including the DCE), and able to provide informed consent. Given Covid-19 pandemic restrictions, participants were also required to have access to a cell phone number so that informed consent and, if necessary, survey interviews could be conducted telephonically. Men were excluded from the study if they were believed to be intoxicated at the time of consent (in which case, they were invited to return the following day), unable to understand the study information, or had previously enrolled in the study.

### Measures and study design

For the “Men’s Choice” study, we used the Theory of Triadic Influence to inform the full selection of measures for the socio-demographic survey in order to understand correlates of HIV-related behavior, focusing on individual, interpersonal, and structural factors relevant for behavior change [52]. For this migration-specific study to understand the role of migration and mobility in this population, questions in the socio-demographic survey included location of birth (South African province or country outside South Africa), duration of time in Gauteng, if and where participants moved in the past two years (between districts, provinces, or countries), reason for moving in the past two years, if applicable, and current residency status in South Africa (permanent resident or citizen, temporary or long-term visa holder, asylum-seeker with or without documentation). We also assessed educational status, relationship status, and employment status. The list of socio-demographic survey measures which we report in this manuscript is shown in Additional file 1: Appendix Table 1. These measures included the frequency of health facility visits; ever testing for HIV and frequency of testing for HIV; self-reported HIV status; and awareness and knowledge of PrEP.

### Statistical methods

We defined internal migrant as South African men born outside of Gauteng Province, and international migrants as men born outside South Africa. We chose to define migrants based on their location of birth to broadly capture men who traveled to Gauteng Province at some point in their lifetime. The International Organization for Migration similarly defines the term “migrant” broadly to encompass “the common lay understanding of a person who moves away from his or her place of usual residence, whether within a country or across an international border, temporarily or permanently, and for a variety of reasons” [53]. We distinguished between men born within and outside of South Africa because these groups may have different HIV and other healthcare experiences in South Africa based on factors such as xenophobia [54], language [19], identification documentation, and citizenship status [28]. We also recognized that internal migrants and international migrants are both heterogenous groups. Therefore, we sought to measure other aspects of migration and mobility, including length of time in Johannesburg, reasons for moving, travel in the past two years, and residency status.

We estimated the prevalence of internal migrants and international migrants by calculating the proportion of each of these categories among all participants. We used Fisher’s exact tests to assess associations between migration factors and HIV and health utilization outcomes. Specifically, we compared self-reported HIV status, HIV testing in the past year, and PrEP knowledge between internal migrants and non-migrants and between international migrants and non-migrants. We used Kruskal–Wallis tests to compare age across different migrant groups and to assess for associations between HIV positivity and age. We used Fisher’s exact tests to compare the proportion of men ever visiting a health facility between South African citizens or permanent residents and men without permanent residency or citizenship. In addition, we used logistic regression to predict HIV status by migration history. For all statistical tests, we assessed significance at the 0.05 level.

#### Ethics

The ethics committees at the University of the Witwatersrand (M191068), Mass General Brigham (Harvard University) (2020P002251), and Boston University (H-40529) approved the study. All participants provided written informed consent. Study data were collected and managed using Research Electronic Data Capture (REDCap), a secure, web-based tool.

## Results

### Socio-demographic characteristics of migrants

In our sample of 150 men, nearly two thirds (62%) were migrants. As shown in Table 1, two fifths (60) of these migrants were internal migrants and one fifth (33) were international migrants. Internal migrants had a median age of 38 years (IQR 32–46 years), and international migrants had a median age of 40 years (IQR 30–42 years). Non-migrants were younger than both internal migrants (Kruskal–Wallis test, *p* = 0.0001) and international migrants (Kruskal–Wallis test, *p* = 0.0012), with a median age of 30 (IQR 25–35). Internal migrants represented all South African provinces outside of Gauteng, with the highest proportion born in Limpopo, followed by KwaZulu-Natal. All international migrants reported being from other countries on the African continent, with three fourths (25/33) from Zimbabwe. Most participants had at least a secondary level of education, although international migrants were more likely to have vocational training (21.2%) as compared to non-migrants (3.5%, *p* = 0.020). Most migrants were in a relationship, but the majority did not live with their primary partner. The majority (85%) of all migrants reported living in Gauteng Province for more than one year, and just under half of all migrants had lived in Gauteng for more than ten years. Only two of the 33 international migrants (6.3%) reported being a permanent resident or citizen of South Africa. Approximately 16% (5/33) international migrants reported holding a visa for South Africa, whereas the remainder of international migrants (78%) reported seeking asylum.

**Table 1:** Recruitment site and socio-demographic characteristics of participants by migrant status (Total *N* = 150)

	**Internal migrants** (*N* = 60)	**International migrants** (*N* = 33)	**Non-migrants** (*N* = 57)	*p-value	**p-value
**Age** Median (IQR, range)	38 (32,46; 20,62)	40 (30,42; 21,73)	30 (25,35; 19,62)	0.0001	0.0012
**Recruitment site** N (column %)				0.000	0.000
Homeless shelter	30 (50.0)	9 (27.3)	23 (40.4)		
Taxi rank	5 (8.3)	0 (0)	19 (33.3)		
Factories	19 (31.7)	6 (18.2)	2 (3.5)		
Public parks	5 (8.3)	2 (6.1)	11 (19.3)		
Building materials establishments	1 (1.7)	16 (48.5)	2 (3.5)		
**Province or country of origin** N (column %)					
	Limpopo 22 (36.7) KwaZulu-Natal 10 (16.7) Eastern Cape 8 (13.3) Free State 7 (11.7) Mpumalanga 7 (11.7) North West 3 (5.0) Western Cape 2 (3.3) Northern Cape 1 (1.7)	Zimbabwe 25 (75.8) Mozambique 4 (12.1) Ghana 1 (3.0) Lesotho 1 (3.0) No response 2 (6.1)	N/A	N/A	N/A
**Education** N (column %)				1.000	0.020
Primary	4 (6.7)	4 (12.1)	3 (5.3)		
Secondary	50 (83.3)	20 (60.6)	48 (84.2)		
University	4 (6.7)	2 (6.1)	4 (7.0)		
Vocational	2 (3.3)	7 (21.2)	2 (3.5)		
**Relationship** N (column %)				0.943	0.123
Living with partner	13 (21.7)	14 (42.4)	13 (22.8)		
Not living with partner	24 (40.0)	12 (36.4)	24 (42.1)		
No relationship	23 (38.3)	7 (21.2)	20 (35.1)		
**Employment** N (column %)				0.246	0.056
Student	2 (3.3)	0 (0)	2 (3.5)		
Employed	4 (6.7)	0 (0)	5 (8.8)		
Piece work	2 (3.3)	6 (18.2)	2 (3.5)		
Self-employed Unemployed	3 (5.0) 49 (81.7)	10 (30.3) 17 (51.5)	10 (17.5) 37 (64.9)		
Retired	0 (0)	0 (0)	1 (1.8)		
**Duration of time in Gauteng** N (column %)				0.000	0.000
< 1 year	11 (18.3)	3 (9.1)	3 (5.3)		
1–2 years	5 (8.3)	3 (9.1)	0 (0)		
3–10 years	15 (25.0)	14 (42.4)	4 (7.0)		
> 10 years	29 (48.3)	13 (39.4)	50 (87.7)		
**Lived outside Gauteng in past two years** N (column %)					
Yes No	11 (18.3) 49 (81.7)	4 (12.1) 29 (87.9)	5 (8.8) 52 (91.2)	0.180	0.720
**Reason for coming to Gauteng in past two years** **(Total *N* = 18)** N (column %)				0.628	0.320
Work	5 (45.5)	3 (75.0)	2 (67.7)		
Education	2 (18.2)	0 (0)	0 (0)		
Family reason	4 (36.4)	1 (25.0)	1 (33.3)		
**Residency status (Total *N* = 147)** N (column %)				1.000	0.000
Permanent resident/citizen	58 (98.3)	2 (6.3)	56 (100.0)		
Asylum seeker with or without documentation	1 (1.7)	25 (78.1)	0 (0)		
Temporary or long-term visa	0	5 (15.6)	0 (0)		

^*^P value for Kruskal–Wallis test (age) or Fisher's exact test (all other variables) comparing internal migrants to non-migrants

^**^P value for Kruskal–Wallis test (age) or Fisher's exact test (all other variables) comparing international migrants to non-migrants

**Fig. 1:**
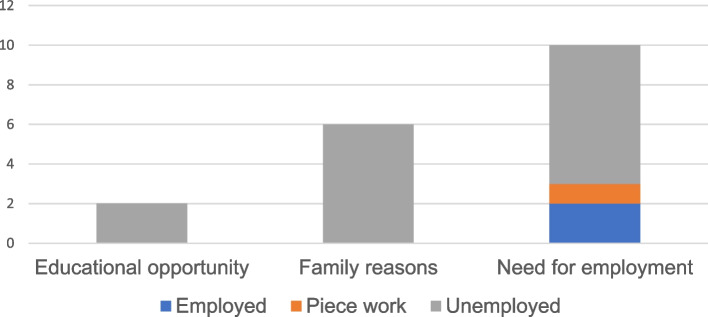
Employment among migrants reporting a reason for moving to Gauteng in the past two years. The vertical axis represents the number of participants for a total of *N* = 18 who responded when asked for their reason for moving to Gauteng in the past two years

Of the migrants who reported on their reason for moving to Gauteng in the past two years (*N* = 18), over half cited seeking or finding employment, followed by family reasons or an educational opportunity. Within this subset of 18 participants, as shown in Fig. 1, 70% migrants who moved to seek employment reported being currently unemployed, and all migrants reporting moving to Gauteng for family or educational reasons reported unemployment as well.

### HIV status

**Fig. 2:**
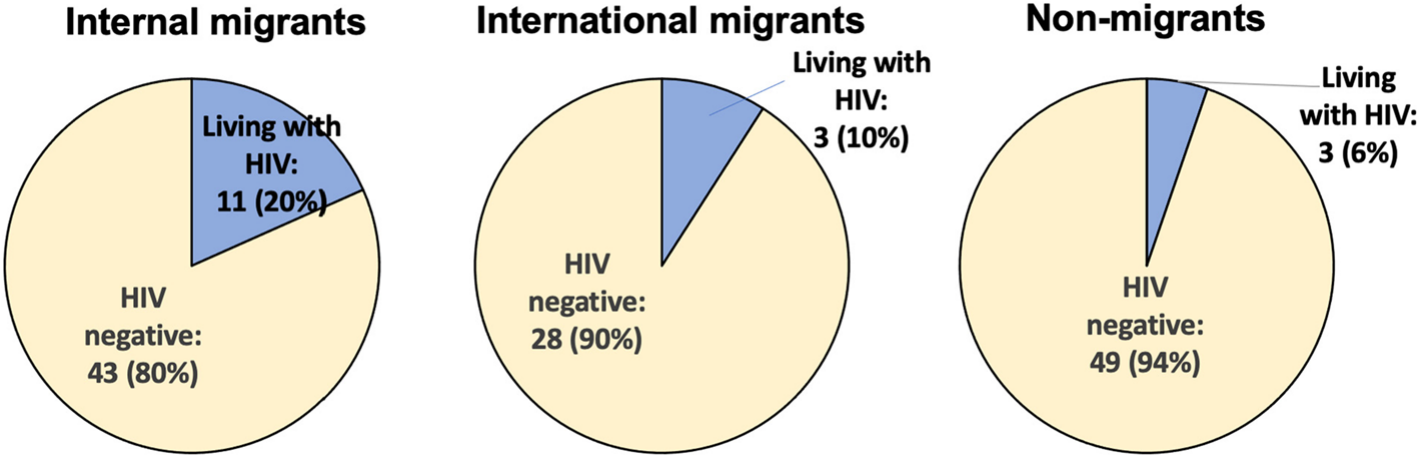
Self-reported HIV status among participants reporting on their HIV status (total *N* = 137). Using a Fisher's exact test to compare HIV status between internal migrants and non-migrants, 20.4% internal migrants who reported on their HIV status (*N* = 54) reported living with HIV, whereas 5.8% non-migrant men who reported on their HIV status (*N* = 52) reported living with HIV (*p* = 0.042). In a logistic regression model controlling for age, this difference in self-reported HIV status between internal migrants and non-migrants was no longer significant

Out of the 137 participants who chose to report their HIV status, 17 reported living with HIV. As shown in Fig. 2, of the 54 internal migrants who reported on their HIV status (90.0% all internal migrants), 11 internal migrants reported living with HIV, representing 20.4% (11/54) internal migrants who reported on their status. In contrast, of the 52 men from Gauteng who reported on their HIV status (91.2% all men from Gauteng), 3 reported living with HIV (5.8%) (*p* = 0.042). A logistic regression predicting HIV status among internal migrants as compared to men from Gauteng resulted in an odds ratio of 4.2 (95%CI = [1.1,16.0], *p* = 0.037). A logistic regression predicting HIV status among internal migrants as compared to men from Gauteng, controlling for age, resulted in an odds ratio of 3.0 (95%CI = [0.7,12.0], *p* = 0.125). Among the 31 (93.9%) international migrants reporting on their HIV status (93.9% of international migrants), 3 (9.7%) reported living with HIV. There was no difference in self-reported HIV status between international migrants and non-migrants. Among the 137 participants reporting on their HIV status, those reporting living with HIV were more likely to be older (median age 42 years) than those reporting not living with HIV (median age 34 years) (Kruskal–Wallis test, *p* = 0.0038).

### HIV care cascade engagement and PrEP knowledge

Almost all participants reported ever testing for HIV (94.6%), with no statistically significant difference in ever testing between non-migrants as compared to either migrant group. (See Table 2) Nearly two thirds of all men tested in the past year, again with no difference between non-migrants as compared to either migrant group. Most men reported “No” or “Unsure” when asked if they had heard of PrEP (84.7%), with similar proportions of internal migrants and international migrants reporting this. When asked what PrEP does, nearly a third of non-migrants answered correctly that it prevents HIV, versus 20.4% internal migrants and 12.0% of international migrants who answered this correctly.

**Table 2:** HIV testing and PrEP knowledge, comparing migrants to non-migrants

	**Internal migrants** **(*N* = 59)**		**International migrants** **(*N* = 33)**		**Non-migrants** **(*N* = 56)**		*p-value	**p-value
	N	% internal migrants	N	% international migrants	N	% non-migrants		
Tested ≥ 1 times for HIV (Total *N* = 148)	55	93.2	31	93.9	54	96.4	0.680	0.625
Tested in past year for HIV (Total *N* = 135)	34	63.0	22	71.0	35	70.0	0.535	1.000
Heard of PrEP (Total *N* = 150)	9	15.0	4	12.1	10	17.5	0.804	0.561
Correct PrEP knowledge (Total *N* = 120)	11	20.4	3	12.0	13	31.7	0.239	0.083

^*^P-value for Fisher's exact test comparing internal migrants to non-migrants

^**^P-value for Fisher's exact test comparing international migrants to non-migrants

### Residency status and overall healthcare utilization

As shown in Table 3, among all men who reported on their migration status as well as their healthcare utilization (*N* = 112), the proportions of internal migrants, international migrants, and non-migrants reporting ever visiting a health facility were similar. Among participants who reported on their residency status as well as their healthcare utilization (*N* = 110), 28.6% men without permanent residency reported never visiting a health facility as compared to 10.6% citizens/permanent residents (*p* = 0.073).

**Table 3:** Healthcare utilization among all participants by migrant status (total *N* = 112) and residency status (total *N* = 110). We used Fisher's exact tests to compare ever having visited a health facility by migrant status and by residency status

	**Ever visited a health facility**		**Never visited a health facility**			
	N	Row %	N	Row %		
					*P-value	**P-value
**Migrant status**					1.000	0.516
Internal migrant (*N* = 42)	37	88.1	5	11.9		
International migrant (*N* = 27)	21	77.8	6	22.2		
Non-migrants (*N* = 43)	37	86.0	6	14.0		
					^α^*P*-value	^β^*P*-value
**Residency status**					0.073	0.385
Asylum-seeker with or without documentation (*N* = 21)	15	71.4	6	28.6		
Permanent resident/citizen (*N* = 85)	76	89.4	9	10.6		
Temporary or long-term visa holder (*N* = 4)	3	0.75	1	0.25		

^*^P-value for Fisher's exact test comparing internal migrants to non-migrants

^**^P-value for Fisher's exact test comparing international migrants to non-migrants

^α^*P*-value for Fisher's exact test comparing asylum-seekers with or without documentation to permanent residents/citizens

^β^*P*-value for Fisher's exact test comparing temporary or long-term visa holders with or without documentation to permanent residents/citizens

## Discussion

In this study of men in Johannesburg, we found that migrants comprised approximately two thirds of our sample, with nearly twice as many internal migrants as compared to international migrants. These proportions are higher than estimates for the proportion of migrants in the province overall, likely reflecting that these recruitment sites, which provide shelter, work, and transit opportunities, attract a disproportionately high number of migrants. Of the participants reporting on their HIV status, a fifth of internal migrants reported living with HIV, while 10% of international migrants and 6% of non-migrants reported living with HIV. Rates of ever testing for HIV were high for all participants, though all participants had low awareness and knowledge of PrEP. Most men reported visiting a health facility, although almost a third of migrants without South African permanent residency or citizenship reported never visiting a health facility at any point in their lifetime.

While our study was not designed to representatively sample migrants within Johannesburg, our finding of a high proportion of internal migrants from Limpopo may support evidence that economic opportunity remains a major driver of migration and mobility within South Africa [38, 55, 56]. Limpopo is South Africa’s fifth most populous province [57], but it has one of the highest rates of poverty in the country [58]. Our high representation of international migrants from Zimbabwe is unsurprising given that there are an estimated one million Zimbabweans in South Africa who migrate for factors including economic opportunities and political unrest in their home country [56]. Within our small sample of men who reported on their reason for moving to Gauteng in the past two years, most moved to Gauteng for employment opportunities. Yet over 80% internal migrants and over half of the international migrants in our sample reported having no current formal or informal (e.g., temporary “piece work” arrangements with payment by unit produced) employment. Despite Johannesburg’s reputation as the “City of Gold,” the economic landscape for migrants remains challenging, with problems including persistent electricity cuts and job losses in the construction, manufacturing, and mining industries [59, 60]. International migrants may face additional hurdles due to new South African laws restricting employment of foreign workers [61]. In addition, Covid-related restrictions during the time of this study further tightened job opportunities and exacerbated vulnerabilities for those working in South Africa’s informal labor sector [62, 63].

It is notable that a small proportion (5/57 or 8.8%) of non-migrants reported living outside of Gauteng in the past two years and that 7/57 (12.3%) of non-migrants reported living in Gauteng for less than ten years. This suggests that although these men are not migrants in terms of their birthplace, they are nonetheless mobile in terms of where they have lived throughout their lifetime. Mobility is associated with heightened HIV risk and poor HIV treatment outcomes due to factors such as interruptions in HIV services and challenges transferring clinics [64]. It will be important for future research to further understand the ways in which mobility and migration overlap in affecting HIV risk and HIV care engagement for men, as well as tease apart the factors that may be uniquely associated with migration or mobility alone.

Our unadjusted analysis found that internal migrant men were more likely to be living with HIV than men from Gauteng. However, this difference in self-reported HIV status was not found to be significant in our logistic regression model when controlling for age. We found that internal migrant men were older than non-migrants, and older men were more likely to be living with HIV. Prior research has also shown an association between older men and internal migration [46] and between older men and HIV infection [65–67]. In addition, prior research has demonstrated an association between migration and higher HIV risk and prevalence [5, 26, 39]. Data show that older men may be less likely to test [68–70] and less likely to know their HIV status as compared to younger men [71]. Given that we relied upon self-report for determining HIV status in this study, our data may have under-estimated the proportion of all participants living with HIV, and particularly for older migrant men. Also, the confidence intervals in our logistic regression models were large, and it is possible that our relatively small sample size limited our ability to detect a significant difference in HIV status between internal migrants and non-migrants. Nonetheless, our findings that 20% of internal migrants and 10% of international migrants reported living with HIV highlight the need for HIV services that are inclusive of migrants’ needs. For example, ensuring sufficient interpreter services to account for the range of languages spoken throughout and outside South Africa may address potential language barriers for migrants in clinical settings [46].

Our finding of high rates of ever testing for HIV among all participants may reflect South Africa’s overall gains in HIV testing [72]. Yet this national progress has been uneven among different populations [73]. Approximately a third of participants had not tested for HIV in the past year, despite the fact that testing at least annually has been shown to reduce the burden of undiagnosed HIV [13], and testing remains an important entry point for engagement in HIV care [74–76]. However, it may be difficult to interpret these data in the setting of Covid-related restrictions, which likely had an impact on HIV testing and other healthcare engagement for all participants during the year preceding this study [62].

Despite overall high rates of ever HIV testing, all participants in our cohort had low awareness of and low knowledge of PrEP. This may be suggestive of general gaps in PrEP awareness [77, 78] and knowledge [79] in South Africa, despite South Africa’s efforts to promote PrEP use through existing services and differentiated service delivery models [80]. In addition, although migrant men are considered a key population in need of targeted HIV services [81, 82], there are few PrEP programs reaching men more broadly [83, 84], and none to our knowledge targeting migrant men specifically. In addition, more than a quarter of men without permanent residency or citizenship reported never visiting a health facility. While it is South African policy that healthcare should be provided in the public sector to all who need it, barriers to healthcare for non-nationals may include fear of exposing one’s undocumented status, stigma for being a non-citizen, or the potential for healthcare fees [28]. These barriers not only impact migrants themselves but may also impede South Africa’s ability to curb its HIV epidemic. For example, neighboring Botswana provides free ART for all citizens, but despite having one of the highest levels of viral suppression globally, HIV incidence remains > 1% per year in adults 15 to 49 [85]. In Botswana, the need to close gaps in persistent HIV incidence has led to calls to expand free ART for all immigrants as well as citizens [85, 86].

Taken together, our findings have important implications for the design of health and HIV programs in South Africa, as well as for other countries with growing rates of migration. In order to close the remaining gaps in reaching UNAIDS targets [87], it is imperative to engage migrant men in HIV services. There is also a need to better understand and address migrants’ knowledge of and attitudes toward PrEP [47, 48] in order to design PrEP programs that include them [81, 88]. Existing research on HIV care for migrants in Johannesburg has focused on international migrants [28, 31], who receive justified national and international attention for challenges that may include xenophobia [89], language barriers, and lack of permanent residency or citizenship status [90, 91]. However, our study highlights that internal migrants also have high rates of HIV and may warrant targeted attention to ensure that they are reached by HIV services.

Our study is cross-sectional and thus limited in that it represents a population of migrant men at one point in time. In addition, our study recruited from community locations in Johannesburg to find men who may not commonly access healthcare services, so our findings may not be generalizable to migrants in other parts of the province or country. We defined “migrant” as born in a province or country outside of Gauteng. This approach, focusing on the spatial dimension of migration, is similar to what has been used in several other studies involving migrants [12, 19, 92]. However, we acknowledge inherent limitations to this approach, as temporal dimensions of mobility (e.g., the amount of time spent in a particular location or seasonal travel) and social dimensions of mobility (e.g., the reason for travel) may contribute to a more nuanced understanding of migrant and mobile populations [7, 34, 39]. We conducted our study in English, isiZulu, Setswana, and isiXhosa, and Sesotho, but we were unable to use all potential first languages for migrants. Response rates were variable for some survey items, such as healthcare utilization, residency status, and reason for migration, which limits our ability to interpret these data. In particular, data on ART use were not obtained due to a data collection error, so we were unable to assess this aspect of care engagement for participants living with HIV. This study focused on HIV and overall healthcare engagement, but future research may consider more deeply the impact of trauma and mental health care as an important need within this population [93]. Lastly, our sample size of 150 participants may not have been sufficient to detect meaningful differences in HIV status and healthcare utilization among migrant and non-migrant populations; future larger studies may detect these differences, if present.

## Conclusions

Our study revealed a high proportion of migrants within our community-based sample of men and demonstrates a need for HIV and other healthcare services that effectively engage migrant men in Johannesburg. Future research is warranted to further explore potential differences in HIV prevalence and healthcare usage among migrant and non-migrant populations, as well as to further disaggregate migrant populations by different dimensions of mobility. We anticipate that our findings, as well as further research among different migrant populations, will have relevance not only for South African’s health system and policies, but also for other African countries experiencing quickly growing rates of migration and urbanization [94].

### Supplementary Information

**Additional file 1: Appendix Table 1. **Key Domains, Variables, and Measures.

## Data Availability

All study data and materials are available upon request by emailing the corresponding author.
