# Supplementary material for: Migrant men and HIV care engagement in Johannesburg, South Africa
Source: BMC Public Health. 2024 Feb 12;24:435. doi: 10.1186/s12889-024-17833-2 (PMC10860300; doi:10.1186/s12889-024-17833-2)
Supplement: Supplementary file 1 — Additional file 1: Appendix Table 1. Key Domains, Variables, and Measures. [file 12889_2024_17833_MOESM1_ESM.docx]

| **Appendix Table 1: Key Domains, Variables, and Measures** | | |
| --- | --- | --- |
| **DOMAIN** | **VARIABLE** | **MEASURE** |
| **Socio-Demographic Factors** | Various measures of socio-demographic factors | Age, gender, level of education, relationship status, employment status |
|  | Various measures of migration-related factors | Adapted from South Africa Household Questionnaire 2009.^91^ Includes Birth location (South African province or other location), duration of time in Gauteng Province, whether lived outside Gauteng in past two years, reason for move in past two years, residency status in South Africa |
| **Healthcare Utilization** | Various measures of healthcare utilization, including for HIV | Internally-constructed  When last visited a health facility, testing for HIV (when tested, frequency, result), knowledge and use of PrEP |
